# Data set on wind speed, wind direction and wind probability distributions in Puerto Bolivar - Colombia

**DOI:** 10.1016/j.dib.2019.104753

**Published:** 2019-11-04

**Authors:** Guillermo Valencia Ochoa, José Núñez Alvarez, Marley Vanegas Chamorro

**Affiliations:** a Facultad de Ingeniería, Grupo de investigación en gestión eficiente de la energía – kaí. Universidad del Atlántico, Carrera 30 Número 8-49, Puerto Colombia, Área Metropolitana de Barranquilla 080007, Colombia; b Universidad de la Costa CUC, Energy Optimization Research Group GIOPEN, Cl. 58 #66, Barranquilla, Atlántico, Colombia

**Keywords:** Wind speed, Wind probability distribution, Wind direction

## Abstract

This paper presents wind speed and direction data measured with a weather station located in Puerto Bolivar, department of La Guajira, situated in the extreme north of Colombia, whose geographic coordinates are 12°11′N 71°55′W. A wind speed and direction sensor, a barometric pressure sensor, and a temperature sensor were used to obtain the presented data. These data were taken at the height of 10 m, which is the highest point of the weather station. The data taken by the meteorological station correspond to a period of 20 years (1993–2013), with hourly frequency. For the missing data, a mathematical model to estimate the Julian averages was developed, allowing to calculate the frequency histograms and four types of probability distributions for these data. Also, the representative wind roses were generated, taking into account the averages in each of the 12 months of the year.

**Table:** Specifications table

Subject área	Renewable energy
More specific subject área	Wind field, wind energy
Type of data	Raw, Graphs, figure, table
How data was acquired	Wind speed sensor Lambrecht Ref. 14521, Wind direction sensor Lambrecht Ref. 12522, Pressure sensor Lambrecht Ref. 8121, temperature sensor Siap + Micros Ref. T001-TTEP-N and relative humidity Siap + Micros Ref. T003-TEH-V
Data format	Raw data and analyzed
Experimental factors	Wind speeds are recorded for twenty years. Both the wind speed and the wind angle are recorded.
Experimental features	Measurements are made at 1:1 scale; sensors are installed to record pressure, temperature, wind speed, and wind angle.
Data source location	Puerto bolívar, Colombia
Data accessibility	Data is with this article

**Table:** 

**Value of the data** • The data provided in this work can be inputs for a projection of future behavior for this region of the country. • The raw data supplied can be used to apply different wind speed and direction calculation procedures to them in future investigations. • The data from the wind direction and speed graph allow estimates to be made of the energy efficiency of wind turbines that can be used in this region.

## 1 Data

The data presented in this paper are meteorological measurements taken at a weather station located in Puerto Bolivar, Colombia. Daily averages of wind speed and direction were obtained. The historical series has some missing data for the measured period. For this reason, in order to complete these data, it was necessary to use an algorithm developed in MATLAB® to calculate the Julian averages for each of the series. This average estimation uses wind speed and wind direction data from the same month, day, and time from other years to calculate the average of these and estimate the missing data.

**Fig. 1:**
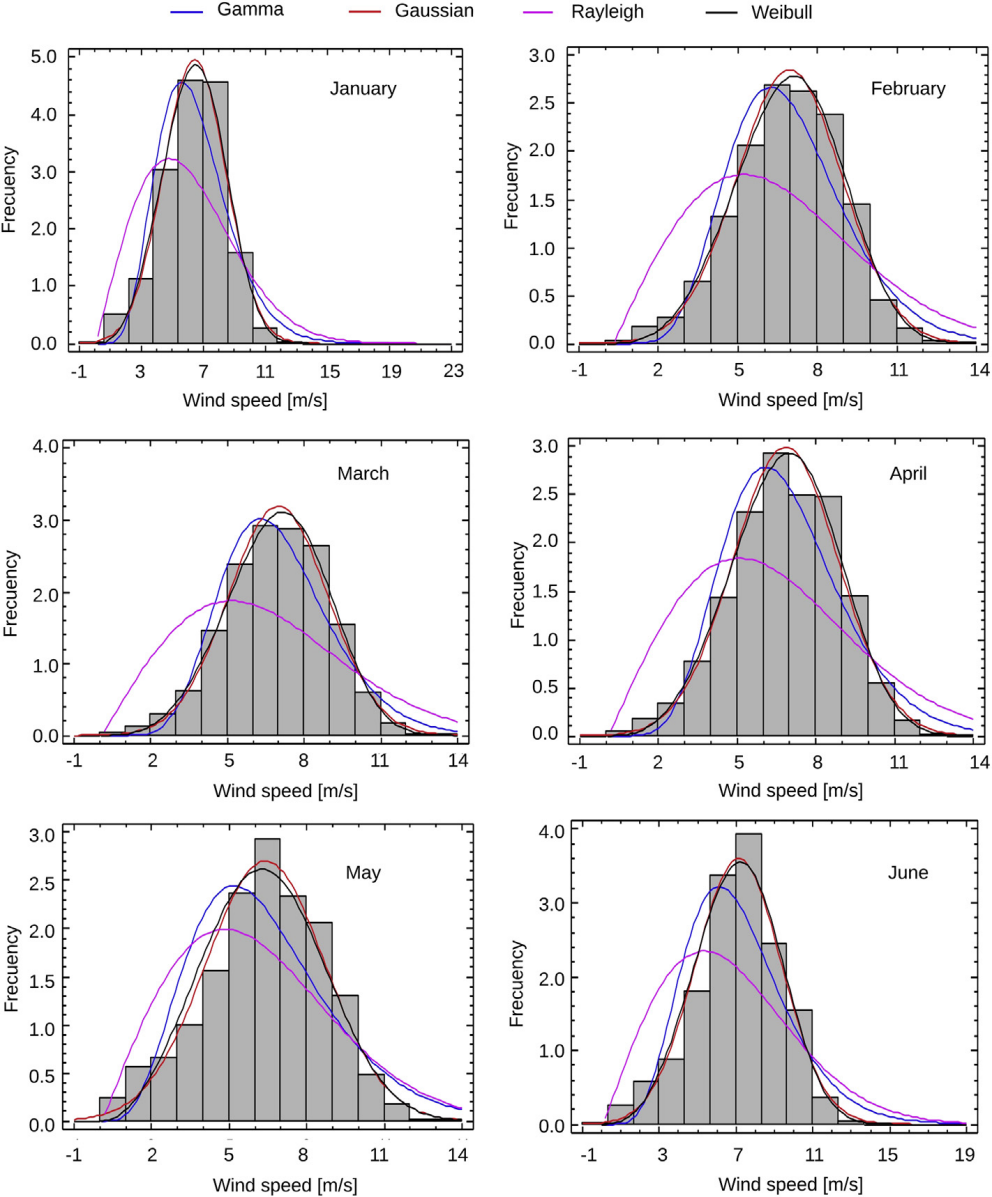
Monthly Probability distributions for Puerto bolivar (January–June).

**Fig. 2:**
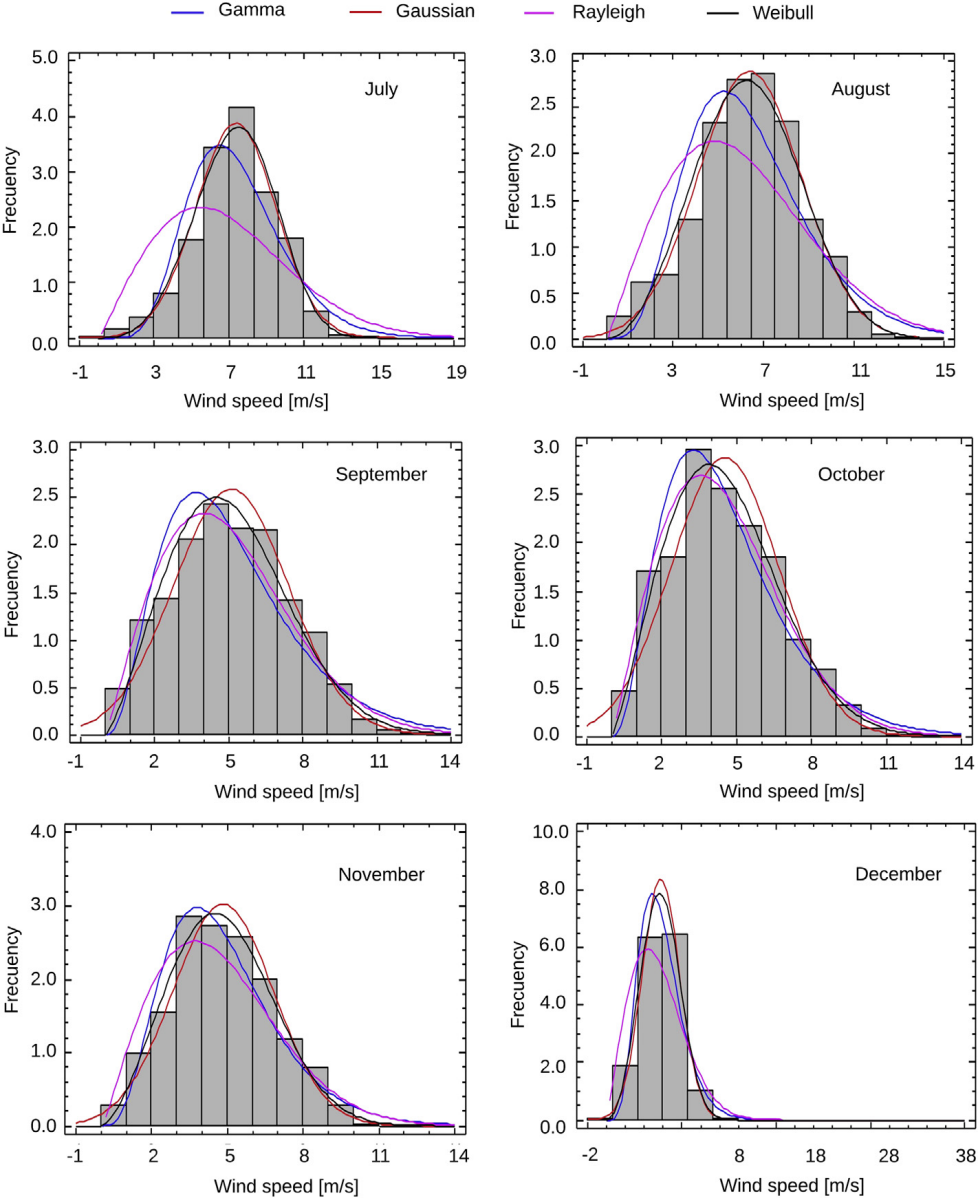
Monthly Probability distributions for Puerto bolivar (July–December).

**Fig. 3:**
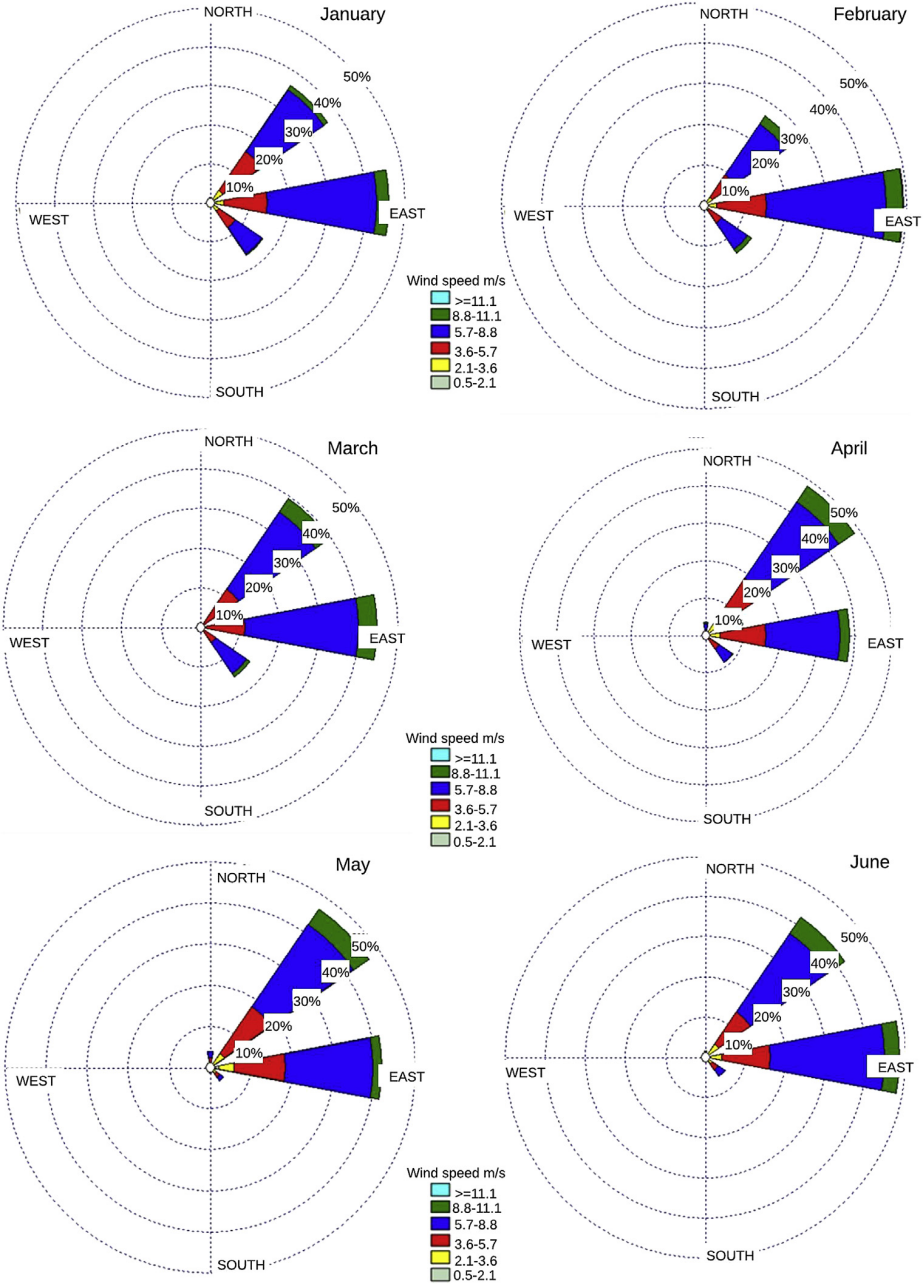
Puerto bolivar wind rose (January–June).

**Fig. 4:**
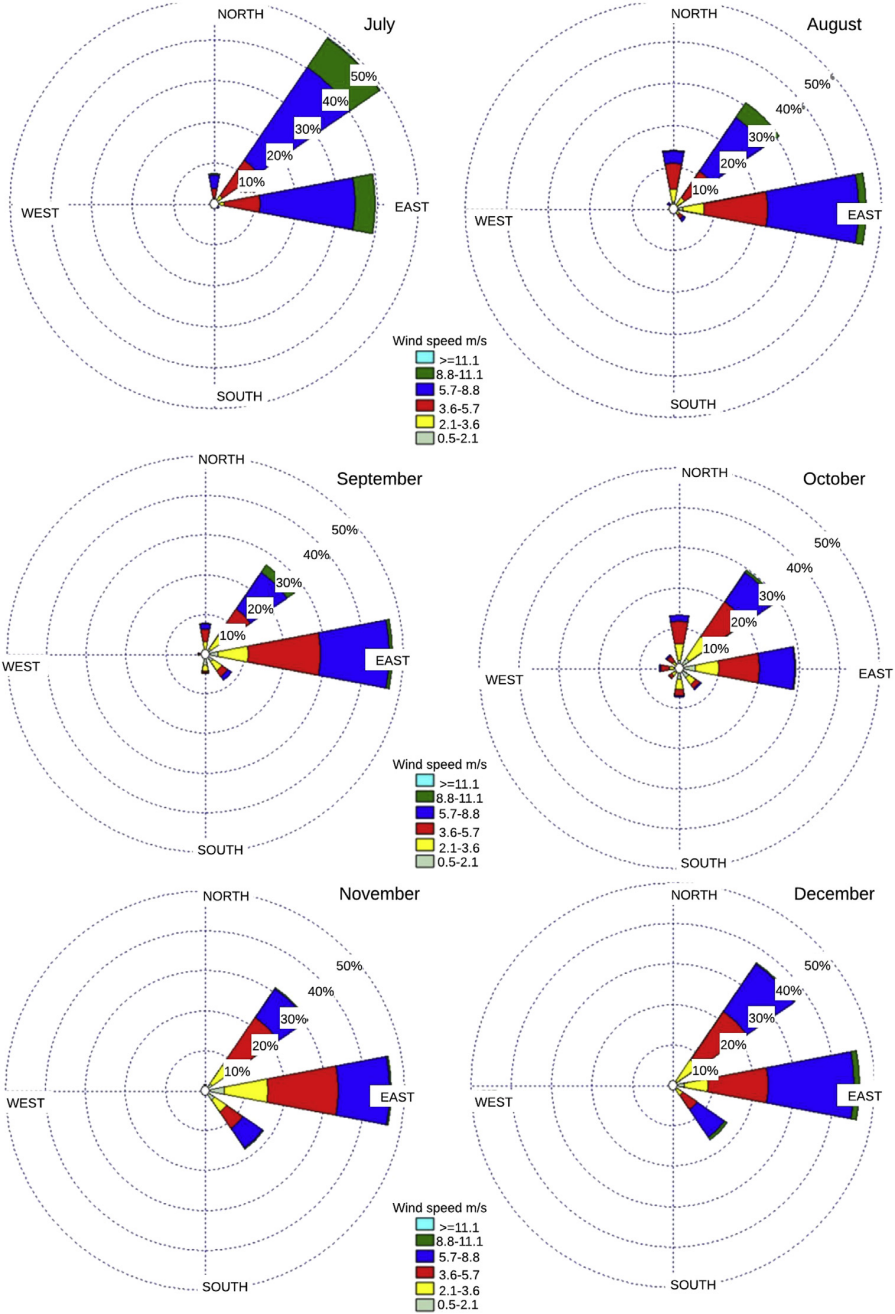
Puerto bolivar wind rose (July–December).

The data series are presented, complemented by different probability distributions that describe the statistical behavior of the data which parameter are shown in Table 1, together with the superposition of four probability distributions obtained from the monthly wind speed and direction data supplied with this document. These distributions are shown in Fig. 1, Fig. 2. In addition, the monthly wind rose graphs are presented in Fig. 3, Fig. 4 to determine the most likely wind direction in the place studied. The original hourly data with which these roses have been generated are presented in the attached documents. The calculations to generate all these figures and tables were made with the data presented in Appendix A.

**Table 1:** Parameters of the Probability distributions.

Month	Gamma		Gaussian		Rayleigh		Weibull	
January	Shape	7.75267	Average	6.42375	Scale	6.63631	Shape	3.61657
	Scale	1.20687	Standard desv.	2.01057	Lower threshold	0.09932	Scale	7.11875
February	Shape	9.74721	Average	6.9427	Scale	6.93962	Shape	3.93514
	Scale	1.40395	Standard desv.	1.99181	Lower threshold	0.29785	Scale	7.65993
March	Shape	10.5084	Average	6.98236	Scale	7.1543	Shape	4.0324
	Scale	1.50499	Standard desv.	1.95163	Lower threshold	0.09938	Scale	7.69446
April	Shape	9.1053	Average	6.84414	Scale	7.04035	Shape	3.8431
	Scale	1.33038	Standard desv.	2.01402	Lower threshold	0.09799	Scale	7.56181
May	Shape	5.31405	Average	3.40028	Scale	6.71382	Shape	3.06432
	Scale	0.83028	Standard desv.	2.30579	Lower threshold	0.09479	Scale	7.13462
June	Shape	7.20571	Average	7.12602	Scale	7.37403	Shape	3.6251
	Scale	1.01118	Standard desv.	2.2335	Lower threshold	0.09835	Scale	7.88605
July	Shape	8.55002	Average	7.35573	Scale	7.57195	Shape	3.88415
	Scale	1.16236	Standard desv.	2.14574	Lower threshold	0.09415	Scale	8.10961
August	Shape	5.6485	Average	6.38453	Scale	6.69418	Shape	3.05505
	Scale	0.88471	Standard desv.	2.29827	Lower threshold	0.09721	Scale	7.12565
September	Shape	3.56666	Average	5.08969	Scale	5.5498	Shape	2.31037
	Scale	0.70076	Standar desv.	2.32575	Lower threshold	0.05068	Scale	5.72938
October	Shape	3.54936	Average	4.53266	Scale	4.9735	Shape	2.20963
	Scale	0.78306	Standard desv.	2.16318	Lower threshold	0.05418	Scale	5.11356
November	Shape	4.68408	Average	4.83386	Scale	5.14798	Shape	2.60483
	Scale	0.96901	Standard desv.	1.99666	Lower threshold	0.08883	Scale	5.43663
December	Shape	6.50515	Average	5.76972	Scale	6.01059	Shape	3.11331
	Scale	1.12755	Standard desv.	1.98683	Lower threshold	0.09653	Scale	6.41973

## 2 Experimental design, materials, and methods

### 2.1 Experiment set up

The weather station was located in Puerto Bolivar, in the department of La Guajira. This department is located in the north extreme of Colombia at coordinates 12°11′N 71°55′W. In this place, was installed a wind speed sensor, wind speed sensor, and pressure sensor, and the parameters of the installed sensors are shown in Table 2.

**Table 2:** Sensors technical data.

Measurement	Range	Precision
Wind speed	0–75 m/s	±0,1 m/s (0,3–10m7s); ±1%(10–55m/s); ±2%(>55m/s)
Wind direction	0–360°	±2% FS
Barometric pressure	600-1100 hPa	±1hPa
Temperature	de 30°c a 60°c	30 to +60 °C; ±0,3 °C
Relative humidity	0-100 %HR	±0,5% RH

**Fig. 5:**
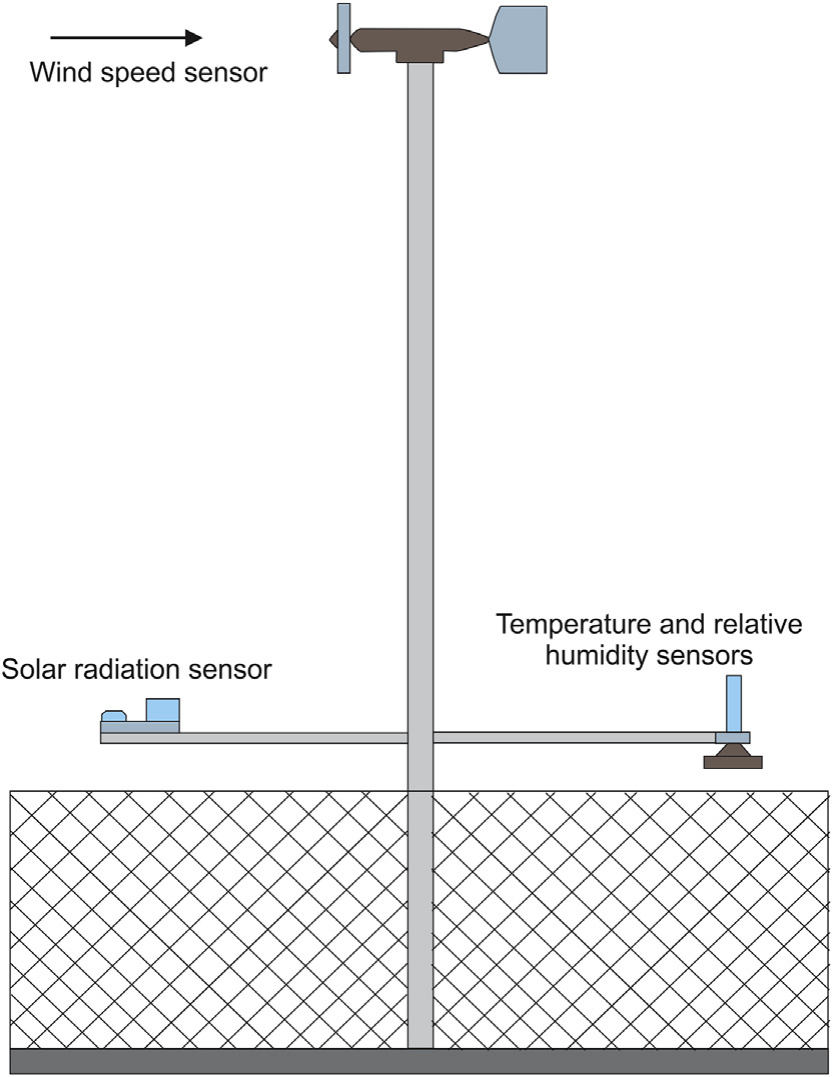
Weather station schematic diagram.

The wind speed and direction were measured at the height of 10 m, this being the highest point of the weather station. A little further down, the temperature and relative humidity sensors were located. The schematic diagram of the weather station used to measure the data presented in Appendix A, is shown in Fig. 5.

### 2.2 Method

**Fig. 6:**
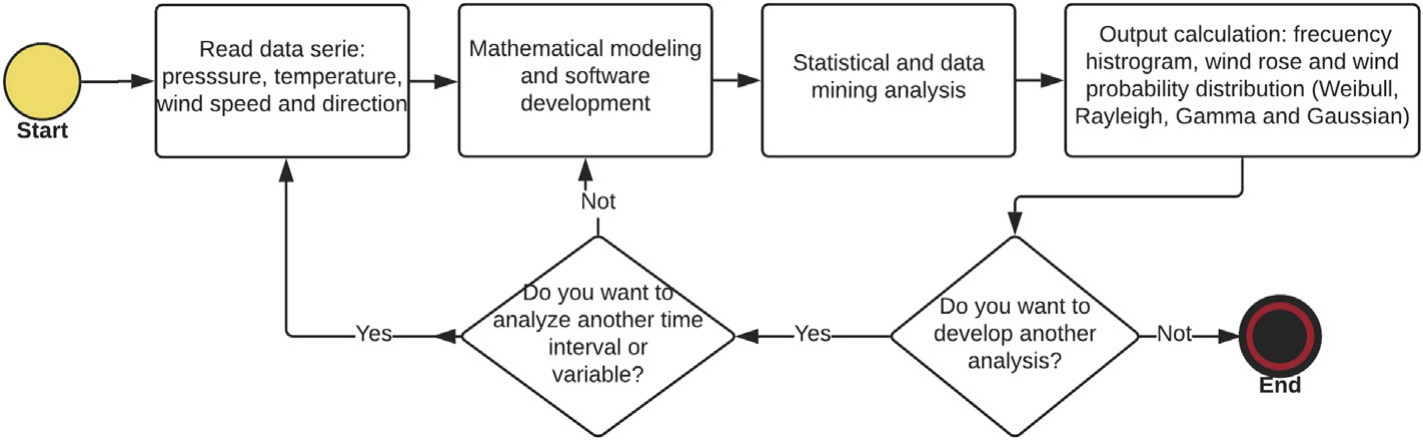
Data analysis flowchart.

The data taken by the weather station correspond to 20 years (1993–2013), with hourly frequency. For the missing data, a data mining was performed to calculate the empty spaces and fill them with the averages from the data on the same day and time in different years, which are presented in Appendix A. Thus, from the totality of these data, four types of probability distributions are calculated. Therefore, the wind speed data are presented as a continuous random variable with an associated probability distribution, and the wind roses give the predominate wind direction presented every month. The methodological procedure developed for the data treatment is shown in Fig. 6, which consists of four fundamental stages.

**Fig. 7:**
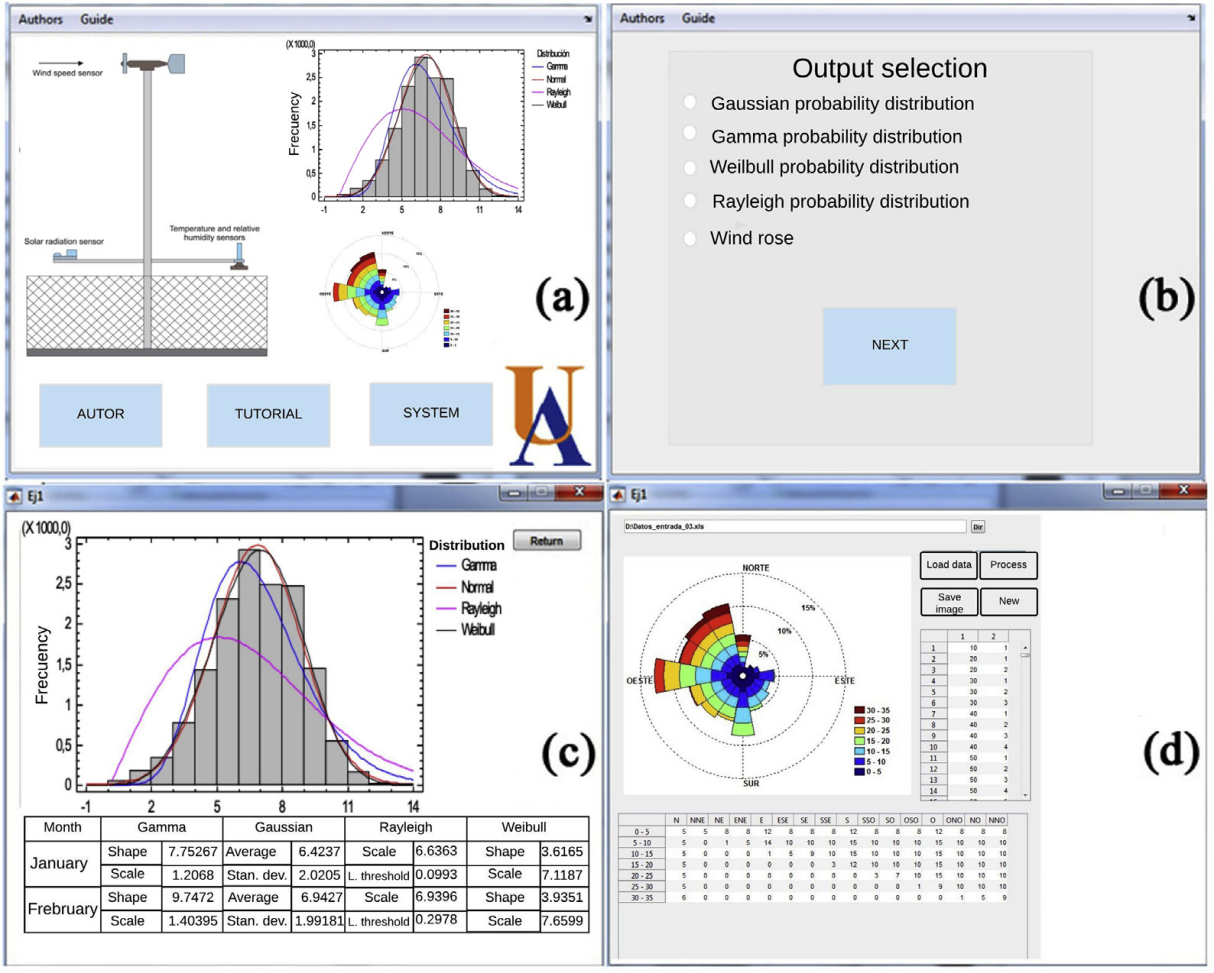
Main view of the WindAnalysisUA v1.0 software, (a) Main view, (b) Output selection, (c) Probability distribution analysis, (d) Wind rose analysis.

In the first stage, the series of data from the sensors installed at the measurement site was read, followed by the development of software for mathematical data processing, allowing the user to select the variables and desired time interval for analysis. Once the variables have been selected, the statistical treatment of the data is performed to calculate the desired outputs, such as frequency histograms, wind roses, and wind speed probability distributions. Fig. 7 shows the main views of the WindAnalysisUA v1.0 software developed to analyze the wind data.

The four probability distributions used were Normal or Gaussian, Gamma, Weibull and Rayleigh [1], which are presented below along with their mathematical models.

#### 2.2.1 Gaussian probability distribution

This distribution can be applied in a large number of case studies, and this makes it a distribution of great statistical relevance [2]. Therefore, this distribution adjusts greatly to physical measurements. This distribution is governed by the probability density function for a normally distributed random variable given by Equation (1) [3,4].

(1) $$f\left(x,\;\mu ,\;\sigma \right)=\;\frac{1}{\sqrt{2\pi }\;\sigma }\exp\left[-\frac{1}{2}\;{\left(\frac{x-\mu }{\sigma }\right)}^{2}\right]$$

where σ is the standard deviation, and −∞<x<∞,−∞<μ<∞ and σ>0.

#### 2.2.2 Gamma probability distribution

This model is commonly used to adjust wind speed distributions. Contrary to the symmetry presented by the Gaussian distribution, the gamma distribution is biased to the right. This function is given by $\text{Γ}\left(\alpha \right)=\overset{\infty }{\underset{0}{\int }}x^{\alpha -1}e^{-x}dx\;for\;\alpha >0$ and the probability density function is calculated using Equation (2) [5].

(2) $$f\left(x\right)=\;\{\begin{array}{c} \frac{1}{\beta^{\alpha }\text{Γ}\left(\alpha \right)}x^{\alpha -1}e^{-x/\beta },\;\;\;x>0\\ \;\;\;\;\;\;\;\;\;\;0\;\;\;\;\;\;\;\;\;\;\;\;\;\;\;\;\;\;\;\;\;\;\;\;\;\;\;\;\;\;\;\; \end{array}$$

where α is the form parameter, and β the scale parameter. When α,β>0, the value of E(X)=αθ, and $Var\;\left(X\right)=\alpha \theta^{2}$.

#### 2.2.3 Weilbull probability distribution

This is another widely applied model for the amplitude distribution of wind speeds over time. This distribution is influenced by the shape parameter (k or α), which varies between values 1 and 3.6. In addition, it depends on a scale parameter (c, θ or β). If a random variable X fits the probability density function expressed by Equation (3), it can be said to have a Weibull distribution [6].

(3) $$f\left(x;\alpha ,\theta \right)=\{\begin{array}{c} \frac{\alpha }{\theta^{\alpha }}x^{\alpha -1}exp\left[-{\left(x/\theta \right)}^{\alpha }\right]\\ 0\;\;\;\;\;\;\;\;\;\;\;\;\;\;\;\;\;\;\;\;\;\;\;\;\;\; \end{array}\;\;\;\;\;\;\;\;\;\;\;\;\;\;\;$$

where x,α,θ>0.

#### 2.2.4 Rayleigh probability distribution

For this probability distribution, there is also a form parameter α and a scale parameter θ, when they take a value of 2 for the form parameter and $\sqrt{2}$ for the scale parameter. The σ parameter is obtained from the probability density function of the Rayleigh distribution, whose mathematical expression is given by Equation (4) [7].

(4) $$f\left(x;\sigma^{2}\right)=\;\frac{x}{\sigma^{2}}\exp\left(-x^{2}/2\sigma^{2}\right)$$

where x>0. In the case in which the shape parameter takes a value equal to 3, one arrives at the Gaussian distribution.

## Conflict of Interest

The authors declare that they have no known competing financial interests or personal relationships that could have appeared to influence the work reported in this paper.
